# Help bring back the celebration of life: A community-based participatory study of rural Aboriginal women’s maternity experiences and outcomes

**DOI:** 10.1186/1471-2393-13-26

**Published:** 2013-01-29

**Authors:** Colleen Varcoe, Helen Brown, Betty Calam, Thelma Harvey, Miranda Tallio

**Affiliations:** 1 University of British Columbia School of Nursing, T149 - 2211 Wesbrook Mall, Vancouver, BC V6T 2B5, Canada; 2 Department of Family Practice, University of British Columbia, British Columbia, Canada; 3 Community Health Representative, Box 463, Bella Coola, BC VOT 1CO, Canada; 4 Family Support Worker, Box 132, Bella Coola, BC VOT 1CO, Canada

**Keywords:** Aboriginal, Rural, Maternity care, Outcomes, Colonialism, Critical ethnography

## Abstract

**Background:**

Despite clear evidence regarding how social determinants of health and structural inequities shape health, Aboriginal women’s birth outcomes are not adequately understood as arising from the historical, economic and social circumstances of their lives. The purpose of this study was to understand rural Aboriginal women’s experiences of maternity care and factors shaping those experiences.

**Methods:**

Aboriginal women from the Nuxalk, Haida and 'Namgis First Nations and academics from the University of British Columbia in nursing, medicine and counselling psychology used ethnographic methods within a participatory action research framework. We interviewed over 100 women, and involved additional community members through interviews and community meetings. Data were analyzed within each community and across communities.

**Results:**

Most participants described distressing experiences during pregnancy and birthing as they grappled with diminishing local maternity care choices, racism and challenging economic circumstances. Rural Aboriginal women’s birthing experiences are shaped by the intersections among rural circumstances, the effects of historical and ongoing colonization, and concurrent efforts toward self-determination and more vibrant cultures and communities.

**Conclusion:**

Women’s experiences and birth outcomes could be significantly improved if health care providers learned about and accounted for Aboriginal people’s varied encounters with historical and ongoing colonization that unequivocally shapes health and health care. Practitioners who better understand Aboriginal women’s birth outcomes in context can better care in every interaction, particularly by enhancing women’s power, choice, and control over their experiences. Efforts to improve maternity care that account for the social and historical production of health inequities are crucial.

## Background

Despite clear evidence indicating that social determinants of health and structural inequities shape health, Aboriginal^a ^women’s birth outcomes may be presented without accounting for historical and current economic and social circumstances. For every indicator of healthy pregnancy and infancy (e. g. teen pregnancy, preterm birth, low and high birth weight, infant and neonatal mortality), outcomes are 2 to 5 times worse for Aboriginal people in Canada, with low birth weight and preterm birth rates worsening [1]. Although research confirms that multiple factors contribute to such outcomes, the social context of women’s lives has been neglected in favor of attention to behaviors (e.g. smoking, diet and prenatal access), psychological factors, immune status, genetic/family history, nutrition, and medical conditions and interventions [2]. However, recent research stresses the mechanisms preceding and underlying these differences. For example, after controlling for income, smoking, and cervicovaginal infection, differences between Aboriginal and non Aboriginal women in low birth weight, prematurity, or macrosomia were statistically non-significant [3]. A study of postpartum depression in single low income women found no differences between pregnant Aboriginal and non-Aboriginal women, pointing to a range of stressors, including income [4]. Similarly, poverty, stress, and low self esteem influenced the relatively high numbers of Aboriginal women who received inadequate prenatal care [5]. Kendall [1] recently argued that:

A long history of colonization, systemic discrimination, the degrading experience of residential schools, and other experiences have led to adverse, multigenerational health effects on Aboriginal families. These experiences have been the root of inequities in the health and well-being of the Aboriginal population, and these inequities have continued through the generations (p. xxxvi).

Increasingly, analyses illustrate how colonizing and racializing processes explain poorer health outcomes, including maternity outcomes, for Aboriginal people in Canada [6-8]. Because studies examining the relationship between chronic psychological stress and pregnancy outcomes typically focus on the 9 month period of gestation, research is required to understand how adverse outcomes may originate long before conception. Emerging literature on allostasis^b^ provides much-needed attention to the cumulative effects of stressors [9] and it may be that low socioeconomic status, racism, exposure to violence, loss, and historical trauma contribute to increased allostatic load and by extension adverse birth outcomes [10,11].

While social determinants of health frameworks increasingly are being integrated in maternity research, and attention is drawn to the specific needs of Aboriginal women, few studies include Aboriginal women or their perspectives. The purpose of this study was to understand rural Aboriginal women’s experiences of maternity care, their desires for future care and what shaped their birth experiences and outcomes.

## Methods

Using a critical ethnographic approach [12,13] within a participatory framework [14], community researchers from Aboriginal communities in Alert Bay, Bella Coola, Old Massett and Skidegate partnered with academic researchers. Ethical approval was obtained from the University of British Columbia (Certificate #H04-80415), and from each of the communities. Our field work drew on both ethnographic and Aboriginal traditions [15,16] so that observations and interviews were conducted in each community observing local protocols and capacity building was considered “a two-way street” [17]. Academic researchers spent time in each community and the entire team met in several times Vancouver and Alert Bay. Community researchers interviewed over 100 women individually or in focus groups. Sampling began purposively with women who had given birth in the previous three years. Women were invited through word of mouth and snowball techniques. Interviews were semi-structured, including questions about the women’s perinatal health care experiences. As early analysis suggested that the perspectives of others would be valuable, particularly men, youth and elders, sampling was expanded to include additional community members through focus groups, informal interviews and community meetings (see Table 1). All individual interviews were conducted by the community based researchers who were members of the local First Nations and trained in ethnographic interviewing by the academic researchers; academic researchers were secondary interviewers in about one-third of the individual interviews and two initial focus groups. All interviews and focus groups were tape recorded and transcribed. All mothers and fathers interviewed identified as belonging to the First Nation in their community, although some were “non-status”^c^. We analyzed data within each community and conducted a cross-community analysis with all members of the team. Transcripts were read individually by each team member within a given community (comprised of two community researchers and two academic researchers per community) and coded into meaning units. Each community team met to identify themes, develop the themes into a conceptual framework and to write a narrative explaining the themes. The community teams then met and compared their analyses, a process which served to challenge and provoke revision of each interpretation, and thus strengthen the rigour of the analysis. Community-specific analyses and recommendations were presented to each community; this paper presents the cross-community analysis.

**Table 1:** Sample and Data collection

**Data Collection Method**	**Sample**
Individual interviews	· 66 mothers
	· 1 father
	· 9 health care /community leaders (e.g. physicians, CEO’s)
Focus groups	· 42 mothers and 2 fathers in 5 groups
	· 11 elders
	· 5 youth (men and women)
Community Meetings	· Small group meetings with health professionals
	· Meetings with cultural centre staff
	· Community meetings
Observations and invited participation at community events (>1000 hours)	· Mother’s and Tots groups and drop ins;
	· Christmas school celebration;
	· Field visit to ancestral village
	· Mother’s Day luncheon with about 60 mothers, fathers and children
	· Baby Welcoming Ceremony
	· Cultural Centre Opening ceremonies
	· Participation in language classes, traditional weaving, salmon preparation

## Results and discussion

**Figure 1:**
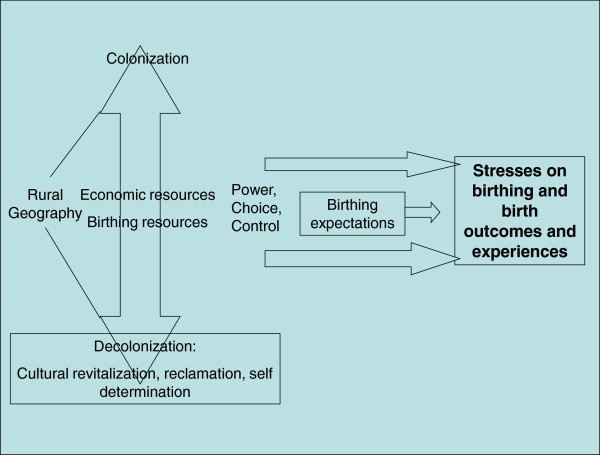
Across Community Analysis of Birthing Experiences in Four Communities.

Across these diverse communities the overarching theme was stresses on birth experiences arising from varied expectations and different levels of power, choice, and control within the context of different resources and histories of colonization and decolonization (see Figure 1: Across Community Analysis of Birthing Experiences in Four Communities). Geography, ease of access, and natural resources available shaped historical patterns of colonization and continue to shape neo-colonial dynamics. Most of these First Nations’ traditional territories are located on remote coastal British Columbia with mountain ranges, storms, fog, steep roads, and great distances between communities. Resources such as fishing and forests have been depleted, leading to high unemployment. As for other First Nations in BC, mineral exploration, resource management practices, and unresolved land claims create conflict. This geography has shaped birthing experiences through the availability of economic resources generally, through the availability of maternity care services, and through challenges to travel.

### Birthing resources

European contact and ensuing colonization decimated traditional birthing practices and Aboriginal midwifery across Canada and imposed Western approaches to maternity care [18,19]. However, following three decades of declining rural maternity services in Canada [20-22] each community was grappling with stresses associated with birthing including dwindling birth and economic resources. Although all communities had some prenatal education and care, there was variation in other services such as ultrasound, transportation for prenatal diagnostic services, and supplementary income for nutrition. While one community had physicians providing full-time “low risk” care, others depended on locums and part-time physicians with varied intrapartum skills. In one community women could give birth in a hospital close to the reserve^d^; in another all women had to leave weeks ahead of their due date to birth in more urban settings. One community had intermittent Caesarean section capacity, but none had reliable access to epidural anaesthesia. The effects of these variations depended on a women’s family and circumstances. For example, women with limited resources associated their postpartum depression primarily with a lack of family and community support before, during and after birth. One said

“*I went through the depression really bad, because I had to be in Vancouver so long by myself…I didn’t know anyone”*.

Women with resources that enabled them to labor and birth with family present connected those experiences to better outcomes.

“*I think it’s better to have [deliveries] in the community, because you can have the whole support of the community and that can help with post-partum depression”.*

Depletion of birthing resources was evident in the almost total destruction of traditional birthing practices in all four communities; only a few surviving elders could recall customs, and this knowledge was being continually lost as elders died. The dominance of the biomedically based health care system, colonizing efforts to eradicate indigenous languages and culture, and the forcible separation of families have hindered efforts to pass on such knowledge in these communities. The extent to which birthing resources beyond the dominant medical system were available varied, and the medical system was constrained by colonial relations between Aboriginal people and the state. Resulting tensions shaped resistance to medical and health care system control of pregnancy and the birthing process.

### Colonial and race relations

Although each community had unique experiences of colonization, there were significant commonalities. The Haida, Kwakwaka’wakw and Nuxalk peoples all suffered devastating population losses from diseases introduced post contact [23,24]. Appropriation and control of resources has affected each community differently. Different levels of affluence shaped power, control, and choice in each community, and the resources available to individual women. For many, very low incomes had direct effects on perinatal nutrition and health, and constrained birthing and “lifestyle” options. For example, without a car or money, many could not get to the nearest town with secondary services to give birth; they stayed in the community regardless of medical recommendations or their own preferences. Those who had “status” and were eligible found the travel allowance was inadequate to eat properly and did not cover bringing their children or childcare. Often the band could not afford an escort (partner or support person).

These dynamics had indirect effects on women’s and families’ sense of power. As a woman in one community described it, *“poverty has no power”.* In another community women were often unable to afford basic nutrition. In contrast, several women from another community appreciated their relative affluence. One said, “*thank God for the Band Council”* referring to the support she received for travel related to a complicated birth.

Racism, the constant companion of colonialism, played out differently in each community. Overt racism was most clearly described in one community. For example, three different women told us that they had overheard health care providers commenting on non-Aboriginal babies’ “tax status”, with one reporting overhearing a provider referring to a Caucasian baby, saying “*at least this is a tax paying baby”*^e^. In contrast, participants from another community made no references to racism regardless of where they gave birth. Those who experienced racism understood such experiences as contributing to stressful relations with health care providers.

### Relations with health care providers

Across the communities there were commonalities in both positive and stressful aspects of women’s relationships with providers in community and referral centres. Positive relations were characterized by respect, understanding of cultural context, and connection with communities.

I just wanted to give birth here, because I knew it would be a better experience just because the doctors and the nurses here know the families more and they’re more relaxed. Like they wouldn’t limit the number of people who were allowed in the birthing room or they would respect whatever wishes I had.

Conversely, negative experiences showed distancing.

“I’m sure I wouldn’t have been as scared if I’d had my own doctor around, to be able to talk to me… the doctors [in the referral centre]… especially the specialists, they kind of looked down their noses at people.”

Relations with individual providers were shaped by the structure and funding of the health care system. In one community women talked about a “revolving door” that created anxiety because they could not develop a relationship with one or two providers:

“I saw a different doctor every month… because the doctors were changing so much here.”

“We both wanted to stay here… but there was… nobody, well no doctors around to actually deliver…”

The structures of funding and availability of resources in one community resulted in induction being used as a scheduling mechanism to accommodate the physician’s schedules, resulting in considerable stress for all women because they did not know until the last minute whether they would need to relocate to give birth. One woman described how she felt being forced to leave her community to give birth to her fourth child and her thoughts about the provider’s roles:

It was heartbreaking… I was mad. I was angry. And it was unfair, I think, to me, for them to make that choice for me. It wasn’t fair at all. And I didn’t want to go. The only way I could stay here was if I got induced. Yeah, that’s what they told me. So, but that was…yeah. So I said, “No thank you, I’d rather go through the natural labouring and everything,” rather than be induced to have him [her son] on their time, and not when he wanted to come out. So, yeah, that’s what I was told. If I wanted him here, I had to be induced that evening, and then just go from there. So I said no. And then, um, they gave me no other choice then, ‘cause the doctor that was here, he was able to perform natural birth, but not a C-section… So, even with my medical record… I had all of them natural… and so I was confident that I was going to have him natural, too. But no, they weren’t confident enough for themselves, I guess.

In that community the women described multiple ways in which what they wanted and needed was overridden: *“I wasn’t allowed an escort”, “I was refused care”, “I was all alone”. “They said, you can be sent out, or you can be induced now… I had a choice, if you call that a choice”*

Tensions characterized many of the women’s birthing experiences, whether they were in local or referral health care centers. One woman who delivered in a referral centre described how dismissive practices affected her:

“The doctor seemed to be in a big hurry. He made it seem like I was inconveniencing him by being in labour at five in the morning. I told him I didn’t want an episiotomy and he gave it to me anyway.”

On the other hand, when women reported positive experiences with providers, it was in contrast to negative experiences, and usually was about what should be considered routine care. For example:

They were, like, bringing me apple juice, and orange juice, and even coffee! I can’t even handle the smell of coffee during labour, so no, thank you. But, yeah, so they were really nice there. I…. it was different from here

When I was in labor I was really scared ‘cause I didn’t know what was happening and I think it was one of the nurses that was so helpful and so nice to me. She showed me what to do and when not to push and when to push.

Thus, relationships with health care providers reflected wider structural issues and social and health care contexts, and shaped the women’s experiences, including a sense of limited power, choice and control, and their birth outcomes.

### Stressful birth

**Figure 2:**
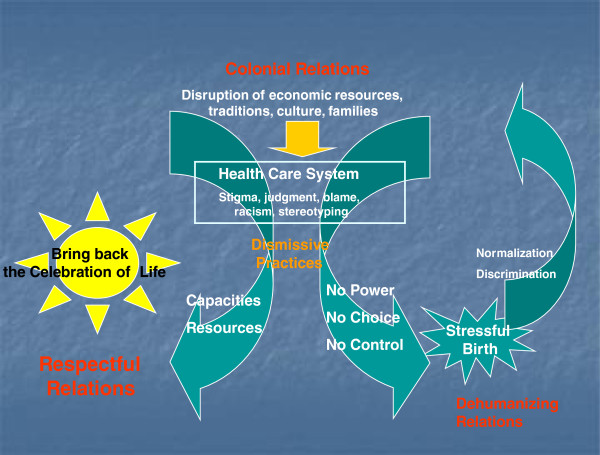
One Community-Specific Analysis of Birthing Experiences.

In all communities women described stresses on birthing specific to rural settings and associated with limited economic resources. In one community, birthing was described as highly stressful by all women interviewed. Here, women described having “no power, no choice, and no control” in their birth experiences and considerable racism in their health care encounters (see Figure 2: One Community-Specific Analysis of Birthing Experiences). They routinely described practices as dismissive. In one focus group all seven young mothers cried through most of the interview, describing the effects of giving birth outside their communities: loneliness, disconnection from community, isolation from family and culture, and discrimination. These were not only short term, ‘acute’ stressors; all seven thought these stresses contributed to their post-partum depression and affected their capacities to bond with and nurture their infants. They also felt distressed and guilty about the children remaining at home while the women gave birth.

It’s hard not to get depressed, it’s hard not to you know feel… stress [being] by yourself and alone and it’s hard to eat when you’re feeling all those things.

Elders who had given birth generations earlier echoed similar concerns. Indeed Elders in one focus group described how their relationships to their children had been disrupted by stressful birth experiences. One woman described how her doctor did not tell her she was having twins, and did not tell her she had to have a Caesarean Section. She then had a harrowing emergency flight to Vancouver, where “the plane literally flipped over because the wind so bad” and the accompanying nurse started to “freak out herself” the situation was so bad. The woman contrasted her relationship with her daughter with her relationship with her sons, attributing the difference to the circumstances of their birth:

I never had the bonds with my sons that I should have had. But I had to work on the bond with my boys. It was a really big difference. Plus having them in Vancouver made it even worse.

### Bringing back the celebration of life

Despite the challenges and stressors, in each community women were motivated, resourceful, and passionate about improving birthing experiences. Community-specific reports with recommendations were developed and presented at community meetings that included Aboriginal leadership, Elders and health care administrators and providers. Researchers from the community in which all births were seen as stressful described their vision as being able to “bring back the celebration of life”. In each community, this involved working toward more respectful relationships, helping health care providers understand history and culture, and strengthening Aboriginal self determination. A teenager in one focus group said:

“…everyone is so happy to go and give to the baby…even if you are not closely related…because it is another member of the Haida Nation, and it just makes the community bigger and richer. In the long run it will make it stronger.”

Within the diverse and unique context of each community, participants identified various strategies for improving birthing experiences. The women’s recommendations varied with their communities, and their individual experiences. Many women were interested in “traditional” and “natural” approaches, although the meaning of natural varied [25]. Some were supportive of the idea of expanding the range of providers, including Doulas and midwives, including Aboriginal midwives, a direction advocated by other researchers [19,26,27] and presently being championed by the National Council of Aboriginal Midwives in Canada (http://www.aboriginalmidwives.ca/). This was particularly the case where women had exposure to alternatives. For example, although midwives were not available in any of the communities, with small numbers of births, and midwifery only recently being re-established in the province, in a community with an active and well-liked doula, this option was frequently suggested by the women. At the same time, having engaged with the language of “risk”, most women emphasized that they wanted access to birth technologies when needed, particularly epidurals and emergency C-sections, with the presence of family, as close to home as possible and within their traditional territories.

Overarching all recommendations, participating Aboriginal women and members of their communities wanted choice and control. They wished to restore a sense of power after centuries of losses in the wake of colonization, and also to move beyond tokenism to authentic participation and leadership in research, planning and decision-making, particularly around issues as central to Aboriginal communities as birthing.

The dominance of biomedicine and the development of western technological interventions in maternity care over the last century have created a situation where Aboriginal communities were told that their time honoured midwifery and birthing practices were unsafe, and that they must turn to the advances of western medical practice for “modern” maternity care. At the same time, community members observed steady erosion in those supposed safety advances to the point where some of their communities are now left with very little of either traditional or western birthing options. Birthing women must undergo further hardships and travel in order to access even the most basic maternity care in a “foreign” environment, or make decisions, sometimes covertly, to take the situation into their own hands and give birth locally against the advice of western medical practitioners.

One of the major stressors for women who participated in this study was the juxtaposition of diminishing local birthing choices, with loss of connection to family, clan and culture when they left their communities to give birth. Many women struggled to come to grips with this, giving examples in interviews and focus groups of negotiating and considering the “risks” inherent in staying or leaving, particularly with respect to disconnection from their people, territory, and traditions. They wondered why medical maternity services were introduced, and then withdrawn from their communities, and who makes these decisions. The struggle itself added greatly to the stresses on birthing. The health impacts of colonization, the effects of medical paternalism, and the struggle to control the bodies of women intersected to magnify the stresses for many rural Aboriginal women, families, and communities. As Couchie *et al.* state, *“Aboriginal women in remote and rural communities should not have to choose between their culture and their safety”*[26].

Our findings reflect Couchie and Sanderson’s [26] recommendations that rural Aboriginal communities and health organizations must collaborate to change existing maternity services, and wherever safe and practical, support the return of birthing to rural and remote Aboriginal communities. What constitutes “practical” and “safe” must be informed by birthing women and their communities. Collaboration must occur in health care interactions at the level of practice, teaching, policy and research levels.

In practice contexts health care providers should pursue culturally safe care by seeking to infuse their own practice and their practice contexts with the knowledge, skills and actions that help them to (1) practice across cultural differences (i.e. biomedical and Aboriginal knowledge of childbirth) and (2) optimize women’s birthing experiences and perinatal outcomes through a recognition of how both are shaped by historical and ongoing colonization, diminished local maternity choices and women’s access to respectful and responsive care that meet their needs. Such practice requires health professional education that will help providers understand how women’s birth experiences, willingness and ability to access care, and perinatal outcomes are shaped by the social, cultural, political, and economic contexts. Participants emphasized that trainees and faculty must gain knowledge of the impacts of colonization, and they would benefit from learning about how specific communities have enacted effective and culturally safe health initiatives. As one of the community researchers said,

“Health care professionals can be guided to be more compassionate to First Nations people and more understanding of our history.”

The overarching implication for policy from this project is that Aboriginal people must have more control over maternity care and birthing experiences. This, of course, echoes the quest of indigenous people globally and throughout Canada to reclaim their lives from colonial intrusion. As reflected in the Canadian National Council of Aboriginal Midwives’ mission statement (http://www.aboriginalmidwives.ca/), the choice of birth place for all Aboriginal communities is consistent with the UN Declaration on the Rights of Indigenous Peoples. Birthing is no ‘small aspect’ of life and health for the communities involved in this study; rather, it is central to culture and wellness. Reclaiming birthing is integral to reclaiming culture and control over people’s own lives. Importantly women and elders must be central to such increasing control. Thus, the findings of this study align with calls for the devolution of control over governance emanating from the Indian Act to Aboriginal people themselves, particularly with respect to economic resources. At the provincial and local levels this project supports the need for increasing control, beyond token representation by Aboriginal people over their economic well-being, and the health care services provided to them. In health care contexts specifically, anti-racist, de-colonizing, and indigenous orientation and training should be required for all employees. Both Aboriginal and non-Aboriginal health care workers can contribute to fostering more respectful, less dismissive, harmful relations. Continued efforts should be made to increase the workforce of Aboriginal health care providers. New models of funding physicians, and new models of care (e.g. doulas and midwives) should be developed *under the guidance* of local Aboriginal people. Health care contexts should be ‘indigenized’, not only by including culturally safe and appropriate care and practices, but by actively taking into account the history of colonization and trauma experienced by Aboriginal people, and the ongoing colonizing practices within Canadian society.

Finally, the findings of this study underscore important directions for both *how* health research should be done, and *what* further research should be done. As we have written elsewhere [17] our experiences in this study support the multiple calls for research that concerns Aboriginal people to be done under the guidance and leadership of Aboriginal people. The next inquiries suggested by this study include action studies and evaluations of new policies, practices and educational strategies. Action studies to enhance rural maternity care for Aboriginal women might take this study as the point of departure. Building on our recommendations regarding practice and policy, collaborative efforts among health authorities, Aboriginal communities and researchers should be undertaken to support the strengthening of birthing to ensure adequate resources are available and to ensure family presence and engagement prior to, during and following birth.

Based on the experiences of women in four Aboriginal communities in British Columbia, this study does not represent the diversity of Aboriginal women in Canada. Nevertheless, the dynamics described suggest directions for understanding health outcomes, practice in relation to Aboriginal maternity care, and further research. This study underscores how socio-political and economic contexts influence birthing experiences and outcomes for rural Aboriginal women. In particular, limited material resources, declining local birthing services and race relations shaped women’s stressors and relationships with providers. Rural Aboriginal women’s birthing experiences cannot be disentangled from how the context of their lives mediates health status in pregnancy and perinatal outcomes. For example, Richmond [28] argues that social determinants such as social support play out in the context of colonialism, and although research has established social support as an important modifiable factor for postpartum depression, the meaning of social support within rural Aboriginal women’s lives is not well understood.

The degree to which the conditions of rural Aboriginal women’s lives create possibilities for positive birth outcomes is obscured when poor birth outcomes are explained on an individual basis. For example, inadequate prenatal care is considered a risk factor for poor birth outcomes, and Aboriginal women have been found to have poorer prenatal care than non-Aboriginal women [5]. Rather than this being a “choice”, our findings show that in addition to geography, prior negative experiences and discrimination were deterrents to accessing prenatal care. If maternity care practices and polices remain oriented to “lifestyle” changes, and social determinants and conditions of women’s lives remain invisible as mediators of outcomes, inequities in Aboriginal maternal-infant health will persist. Accounting for history and culture can generate new insights for maternity care tailored to local contexts.

What is *not* measured as a significant mediator of women’s experiences and birth outcomes also is important. In this study the erosion of birthing and economic resources fostered negative birth outcomes and experiences. Disconnection from community fractures social supports, including family relationships and cultural practices; thus the structural and funding conditions that compel women to leave their communities warrant analysis and action to mitigate poor birth outcomes. Klein at al. [29] describe how erosion of local birthing capacity creates a “cascade of adverse consequences” for mothers, babies, and entire communities. Although not focusing on Aboriginal communities, Klein et al. show how effective maternity and newborn care is fundamental to the social and economic sustainability of communities. Emerging evidence about allostatic load in pregnancy and the results of research examining social determinants of birth outcomes, suggest that it is necessary to (1) better assess the structural conditions and mechanisms by which they produce health outcomes and (2) build upon such assessments to generate outcome measures sensitive to the conditions within which outcomes are produced. Most importantly, the structural inequities that produce disproportionately poorer health for Aboriginal people (e.g. poverty, racism, inferior educational opportunities) must be redressed. Aboriginal communities must have control over decisions affecting their health and health care, including maternity services.

## Conclusion

Historical and ongoing colonial relations impact birthing resources and experiences for Aboriginal women. Addressing the mechanisms through which stresses influence birth outcomes will require interventions in policy and practice. Beyond modifying individual lifestyle behaviours, actions are required to diminish stressors arising from racism, poverty, and the organization, funding, and delivery of rural care. Individual providers can improve care for Aboriginal women by learning and accounting for community and family history, and by enhancing women’s control over their experiences. Indicators of maternal and infant outcomes require expansion to encompass intermediate mechanisms that support or degrade health. Policies that account for the social production of health inequities can also improve maternity care – for example by mitigating the effects of poverty, and housing and food insecurity. While initiatives to bring birthing closer to communities are important [26], broader approaches are required and health care providers can align with communities and women to promote such change.

## Endnotes

^a^In Canada, the term “Aboriginal people” refers to indigenous peoples and encompasses First Nations, Métis and Inuit peoples [30]. These three groups reflect ‘organic political and cultural entities that stem historically from the original peoples of North America, rather than collections of individuals united by so-called ‘racial’ characteristics’ [30](p. xii).

^b^Allostasis is a more precise alternative to the term *stress,* used to refer to environmental challenges that cause an organism to begin efforts to maintain stability (allostasis).

^c^“Status” refers to those defined under the Indian Act as “status Indians” who are then governed by the Act

^d^“Reserve” is the term used in Canada to refer to lands set aside for the use of Aboriginal people under the Indian Act of 1867 [31]

^e^A particularly pernicious and pervasive race-based discourse in Canada contends that because “status” First Nations people do not pay the same taxes as other Canadians they “get everything for free”. This discourse is often applied a) regardless of whether the people to whom it is being applied are “status” or not, b) without understanding that most Aboriginal people in Canada pay most taxes, c) without acknowledging that the land on which they do not pay tax is but a fraction of their traditional territories appropriated through colonial conquest.

## Competing interests

The authors declare that they have no competing interests.

## Authors’ contributions

CV and BC co-led the overall research. HB served as the research coordinator for the overall research. TH and MT were community based researchers, conducting all individual interviews and co-conducting all focus groups in their community, and leading their community-specific analysis, report writing and community presentations. All authors participated with the larger team in the cross-community analysis, and contributed to the research report upon which this article is based. All authors read and approved the final manuscript.

## Authors’ information

This research has been followed up in diverse ways specific to maternity care and more generally. The 'Namgis community researchers and author HB have continued to work together to examine how tradition and culture have protective and health promoting impacts that are indigenizing approaches to health policy and planning within Alert Bay. Community members in Old Massett initiated and continued baby-naming ceremonies. Author BC has continued to collaborate with the Haida communities. Author CV has been involved in research to improve primary health care for Aboriginal people, for example using the Provincial Health Services Authority’s “Indigenous Cultural Competency” (an antiracist training for health care providers) as part of an organizational level intervention to promote equity and social justice and improve care and health outcomes.

## Pre-publication history

The pre-publication history for this paper can be accessed here:

http://www.biomedcentral.com/1471-2393/13/26/prepub
